# Diabetes Mellitus and Gastric Cancer: Correlation and Potential Mechanisms

**DOI:** 10.1155/2023/4388437

**Published:** 2023-11-09

**Authors:** Li Wang, Zhe Zhang

**Affiliations:** ^1^Department of Emergency, The Second Affiliated Hospital of Zhejiang University School of Medicine, Hangzhou 310052, China; ^2^Zhejiang Provincial Critical Research Center for Emergency Medicine Clinic, Hangzhou 310052, China; ^3^Key Laboratory of Diagnosis and Treatment of Severe Trauma and Burn of Zhejiang Province, Hangzhou 310052, China; ^4^Department of Emergency Medicine, The First People's Hospital of Linping District, 311100, Hangzhou, Zhejiang, China

## Abstract

This review summarizes the correlation between diabetes mellitus (DM) and gastric cancer (GC) from the perspectives of epidemiology, drug use, and potential mechanisms. The association between DM and GC is inconclusive, and the positive direction of the association reported in most published meta-analyses suggests that DM may be an independent risk factor for GC. Many clinical investigations have shown that people with DM and GC who undergo gastrectomy may have better glycemic control. The potential link between DM and GC may involve the interaction of multiple common risk factors, such as obesity, hyperglycemia and hyperinsulinemia, *H. pylori* infection, and the use of metformin. Although *in vitro* and *in vivo* data support that *H. pylori* infection status and metformin can influence GC risk in DM patients, there are conflicting results. Patient survival outcomes are influenced by multiple factors, so further research is needed to identify the patients who may benefit.

## 1. Introduction

Diabetes mellitus (DM) is projected to affect 500 million people worldwide by 2030 [1]. Gastric cancer (GC) is a lethal tumor that affects the digestive system. It is the fifth most frequent cancer and the fourth major cause of cancer mortality in the world. There are nearly one million new GC cases and nearly 700,000 deaths worldwide per year [2]. However, despite a vast literature on the relationship between DM and cancer, the association with GC varied in different studies. Therefore, this review summarizes the correlation between DM and GC from the perspectives of epidemiology, drug use, and potential mechanisms.

## 2. Epidemiological Analysis of DM and GC

DM is a metabolic disorder characterized by insulin malfunction that is often accompanied by severe consequences, such as hyperglycemia, hypoinsulinemia, and insulin-like growth factor- (IGF-) related metabolic dysfunction [3]. DM increases the risk of certain cancer types, such as pancreatic cancer [4], breast cancer [5], endometrial cancer [6], and colorectal cancer [7] and increases mortality from any cancer [8]. DM is present in 8% to 18% of all cancer patients. Compared with nondiabetic patients, diabetic cancer patients have a 42% increased risk of death and a 21% increased risk of tumor recurrence [9]. Although epidemiological studies have shown that DM has a certain impact on gastrointestinal tumors [10], the association between DM and GC is still controversial.

### 2.1. DM Increases the Risk of GC

An analysis of 80,193 gastrointestinal cancers from five European and three Asian countries revealed that the overall prevalence of DM was 14.8% (11,866/80,193). Among them, the prevalence of DM was highest in colon and rectal cancer patients (15.5% vs. 15.3%, respectively) and 14.0% in GC patients, both of which were significantly correlated with the high incidence of DM [11]. A meta-analysis also revealed a statistically significant relationship between DM and GC incidence (RR = 1.11, 95% CI: 1.00-1.24, *P* = 0.045, *I*^2^ = 79.5%) [12]. The results of a large retrospective cohort study conducted in Korea that included 195,312 patients who had a more accurate diagnosis by endoscopic examination revealed that individuals with DM were at an elevated risk of developing GC (estimated adjusted HR = 1.76, 95% CI: 1.04-2.97) [13].

### 2.2. DM Does Not Increase the Risk of GC

A meta-analysis of 22 cohort studies and 8,559,861 participants found that DM had little or no change in the risk of GC [14]. There was no evidence of a significant difference in the RR for GC between men and women (RR = 1.10, 95% CI: 0.94-1.29, *I*^2^ = 22.9% in men; RR = 1.00, 95% CI: 0.90-1.11, *I*^2^ = 97.2% in women) [14]. Compared with normal blood glucose participants, the risk of GC was not increased among participants with prediabetes (HR = 1.07, 95% CI: 0.79-1.44), DM (HR = 0.77, 95% CI: 0.46-1.29), or any of these exposures (HR = 0.96, 95% CI: 0.73-1.27) [15]. The relationship between DM and GC is unclear based on previous epidemiological studies. A two-stage individual participant data meta-analysis including 5,592 cases of GC and 12,477 controls from 14 studies from North America, South America, Europe, and Asia did not find an association between DM and GC (pooled OR = 1.01, 95% CI: 0.94-1.07). However, the risk of gastric cardia cancer was significantly higher with T2DM (OR = 1.16, 95% CI: 1.02-1.33) [16].

### 2.3. Whether DM Increases the Risk of Death from GC

DM can disrupt the body's immune function and metabolic processes [17], leading to disturbances in energy balance that may impact the development and outcomes of cancer [18]. However, it is important to note that specific prognostic outcomes can vary from individual to individual, depending on factors such as DM management, treatment modalities (e.g., surgery and adjuvant chemotherapy), and antidiabetic medications such as insulin [19].

There are some studies that suggest that patients with DM may face a higher risk and lower survival rate in GC treatment [11–13]. Studies showed that high fasting blood glucose (≥126 mg/dL; RR = 1.09) increases the risk of GC [20]. The blood glucose variability in GC patients is significantly higher than that in non-GC patients, and higher blood glucose variability in patients without DM will also increase the risk of GC [21]. Preoperative metabolic syndrome, particularly hyperglycemia, predicted GC mortality in patients receiving radical gastrectomy, particularly in patients with early GC, according to a sizable cohort research by Hu et al. [22]. However, other studies have not observed this association. According to Miao et al., Zheng et al., and Dabo et al., there has not been much of a difference in the death rate or risk of getting GC in DM individuals [14–16]. In addition, Bae's meta-analysis of prospective cohort studies found no evidence linking a history of DM to an increased risk of GC [23].

Differences in study populations, exposure assessments, lengths of follow-up, and adjustment for confounders might explain the high degree of heterogeneity in the findings. In particular, the exposure assessments and duration of follow-up varied considerably across studies. Furthermore, confounding variables such as gender, age, BMI, population, race, culture, lifestyle, environment, and socioeconomic position will alter the incidence of diabetes or GC and may even raise the risk of GC among people with diabetes [24]. Significant gender and geographical disparities in the prevalence of type 2 diabetes mellitus (T2DM) and GC have emerged over the last 30 years, suggesting complicated links with race, immigration, culture, lifestyle, gene-environment interactions, socioeconomic level, and social role inequalities [25]. The influence of genetic effects, epigenetic processes, dietary variables, and lifestyle on the risk and result of T2DM and GC development differs between men and women [26]. Sex hormones influence insulin sensitivity and secretion, stomach emptying and glucose absorption, vascular function, energy metabolism, and inflammatory response in women with excess androgen or males with impaired gonadal activity [27]. GC has a significant male advantage, and greater levels of circulating dehydroepiandrosterone may be related to a decreased risk of noncardiac GC [28]. In addition, there is a link between blood levels of androgens, estrogen, and sex hormone-binding globulin in males with the chance of developing primary GC [29].

Furthermore, physiological and psychological variables contribute to gender variations in T2DM and GC risk and prognosis [30]. However, there is currently a scarcity of randomized controlled studies that show gender-specific benefits using well-designed intervention measures. Gender differences must be studied using appropriate animal models and translational research to better understand the pathophysiology and complicated interplay of hormones, genes, lifestyle, and environment in T2DM and GC patients. As a result, the effect of DM on the risk of GC or death must be explored further. Understanding the possible impact of DM on GC risk is a critical component of DM treatment.

### 2.4. Remission of DM after Gastrectomy

Many clinical investigations have shown that people with DM and GC who undergo gastrectomy may have better glycemic control [31, 32]. T2DM remission rates vary from 42.5% to 65.4% in patients with GC following gastrectomy (Table 1). A meta-analysis of 11 randomized controlled trials provided class 1A evidence demonstrating that patients who undergo bariatric surgery experience T2DM remission [33]. After gastrectomy, insulin resistance is shown to decrease, and fasting glucose returned to normal; however, the cause of remission is still unknown [34]. According to An et al.'s study, the length of T2DM remission was substantially associated with the degree of remission [35]. Kim et al. concluded that BMI reduction was significantly associated with remission of T2DM [36]. Total gastrectomy with RY reconstruction has a greater remission rate than other surgical procedures; however, it is unclear whether the scope of the gastrectomy or the manner of reconstruction has a role in T2DM remission. According to Wang et al., the degree of gastrectomy, rather than the method of reconstruction, was the most important factor determining T2DM remission [37]. On the other hand, Choi et al. found that RY reconstruction is crucial for T2DM remission [38]. A meta-analysis by Peng et al. suggested that only the degree of gastrectomy can affect T2DM remission [39], which may also affect overall survival. Wei et al. found that recovery from preexisting T2DM following radical gastrectomy was highly related to higher overall survival in a sample of 67 patients [40]. Although the mechanism of T2DM remission following gastrectomy is unknown, bariatric surgery may promote T2DM remission by promoting lifestyle modifications, including reduced food intake, weight loss, and intestinal malabsorption. In addition, there are a few theories that might explain why hyperglycemia improves following gastrectomy. The foregut theory states that resection of the duodenum and proximal jejunum may prevent the secretion of some signals that promote insulin resistance, but this signal is still unknown and remains less well proven in human subjects [41]. According to the hindgut hypothesis, rapid transport of unabsorbed nutrients to the distal intestine might boost intestinal hormone release [42]. In addition, ghrelin, another gut hormone that stimulates appetite and food intake, is mainly produced by gastric X/A cells, and its level is reduced after gastrectomy [43]. Changes in gut microbiota following the Billroth II or Roux-en-Y gastric bypass have also been linked to DM remission and improved metabolic control in two recent investigations; the main manifestations were reduced incidence of metabolic syndrome and T2DM and increased postoperative intestinal microbial richness and diversity [44, 45].

## 3. Potential Mechanisms between DM and GC

**Figure 1:**
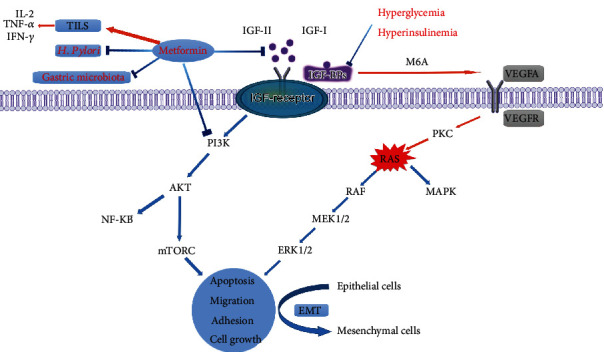
Potential mechanisms between DM and GC. Abb: IL-2: interleukin-2; TNF-*α*: tumor necrosis factor alpha; INF-*γ*: interferon-gamma; TILS: tumor-infiltrating lymphocytes; IGF: insulin-like growth factor; IGFBP: insulin-like growth factor binding protein; VEGF: vascular endothelial growth factor; EMT: epithelial-mesenchymal transition.

Although the connection between DM and GC is yet unknown, numerous biological theories have been postulated, including obesity, hyperglycemia and hyperinsulinemia, *Helicobacter pylori* (*H. pylori*) infection, and the use of certain medications (e.g., metformin) [16, 46–49] (Figure 1).

### 3.1. Influence of Obesity on DM and GC

Multiple meta-analyses suggest that obesity and unhealthy lifestyles may have deleterious effects on GC risk [50]. The prevalence of DM significantly increased from 1995 to 2014, and associations between DM and obesity are well established [51]. Obesity is associated with insulin resistance, compensatory hyperinsulinemia, metabolic syndrome, and T2DM. Overweight or obesity is linked to a higher risk of GC, and the intensity of this link is stronger as BMI rises, particularly in Asian populations [52]. Rawla and Barsouk suggested that DM patients were more likely to suffer from obesity and gastroesophageal reflux disease, leading to a significant increase in the risk of gastric cardiac cancer [53]. On the other hand, Lin et al. found no change in the incidence of stomach cardiac carcinoma attributable to DM across BMI strata [54], suggesting that other factors unrelated to obesity may be involved in the pathogenesis of gastric cardiac cancer. Further studies are needed to prove these hypotheses.

### 3.2. Effects of Hyperglycemia and Hyperinsulinemia on DM and GC

At the molecular level, *in vitro* and *in vivo* studies show different mechanisms for hyperglycemia and hyperinsulinemia leading to the development of GC, such as increased cell proliferation, promotion of angiogenesis, oxidative DNA damage and overstimulation of tumorigenic pathways [55]. Hyperglycemia has been linked to tumor vascularity, metastasis, and the expression of vascular endothelial growth factor in many investigations [56]. Hyperglycemia can lead to DNA damage directly or can damage through the production of reactive oxygen species (ROS). Metabolism-induced oxidative stress may promote epithelial mesenchymal transformation, leading to the accumulation of tumor genes and tumor suppressor gene mutations, promoting gastric mucosal damage and interfering with repair [13]. Furthermore, hyperglycemia can generate more energy through glycolysis and lactic acid pathways, leading to energy balance imbalance, affecting intracellular metabolism and damaging immune function, complement activation, and antioxidant systems [57]. Patients with DM may have increased susceptibility to *H. pylori* infection and delayed wound healing after infection due to immunosuppression caused by hyperglycemia [58].

Hyperglycemia can also significantly trigger insulin secretion, and hyperinsulinemia can overactivate insulin signaling. Furthermore, chronic hyperglycemia may also cause an increase in the formation of ROS and oxidative stress [59], both of which are thought to promote carcinogenesis and cancer development [60]. Additionally, insulin resistance in DM promotes inflammation and activates nuclear factor-*κ*B (NF-*κ*B), which is a light-chain enhancer of activated B cell signaling that plays a major role in GC development and progression [61]. Hyperglycemia can also provide more glucose to tumor cells, promote tumor proliferation and migration, and activate GC cells to migrate to lymph nodes [62]. In addition, abnormal fluctuations in glucose levels in DM patients also increase oxidative stress, endothelial dysfunction, and subclinical inflammation. NADPH oxidase activity in mitochondria induces superoxide production [63], and the AKT signaling pathway is inhibited by increased NF-*κ*B and caspase-3 expression [64]. Therefore, fluctuations in fasting glucose are considered to be linked to an increased risk of GC [21, 65]. Plasma insulin levels were also positively associated with GC compared with hyperglycemia [66].

Hyperinsulinemia leads to elevated levels of insulin-like growth factor 1 (IGF-I), a potent pro-mitogen that can cause cancer and decrease apoptosis in cancer cells [67]. Hyperinsulinemia may also overstimulate tumorigenic pathways, such as IGF-II/IR-A signaling, which is thought to be a key promoter of cancer in people with diabetes or prediabetes [66, 68]. Insulin receptor (IR), IGF-I, and IGF-II are all significantly expressed in GC cells, and the IGF-I/IGF-IR pathway has been considered an important therapeutic target for GC [69]. The fact that GC cell survival is dependent on IR but not the IGF-I receptor suggests that IGF-I/IGF-II increase GC cell survival through IR [70]. Hyperinsulinemia and overexpression of IGF can activate the mitotic pathway or stimulate tumor growth by inhibiting the expression of IGF-binding proteins (IGFBPs), which play a key role in the carcinogenesis and metastasis of GC [71]. Hyperinsulinemia may also downregulate IGFBP levels, indirectly leading to elevated levels of IGF [72]. In addition, insulin is a mitogen and cell survival factor expressed in almost all cell types that activates signal transduction, stimulates cell growth, and promotes cell survival and is considered to be a potential mechanism for the association between DM and cancer [55]. Saisana et al. confirmed that high expression of the insulin receptor can be detected in metastatic GC cells and cell lines, which can stimulate PI3K/Akt signal transduction, cell proliferation, and the survival of GC cells [73]. Knockdown of the insulin receptor can inhibit tumor cell proliferation and induce programmed cell death. These results suggest that insulin and insulin receptors can synergistically promote the occurrence and development of GC. Some studies have also found that *H. pylori* infection can potentially disrupt the balance of gastrointestinal microbiota, consequently impacting energy metabolism and insulin sensitivity in the body. This disruption may lead to insulin resistance, where the cells become less responsive to insulin, ultimately developing hyperinsulinemia [74]. Previous studies have also confirmed that insulin use in DM patients is significantly associated with a high incidence of *H. pylori* eradication [75].

### 3.3. Effects of Biomarker on DM and GC

IGFBP, IGF-I, and numerous growth factors, including vascular endothelial growth factor (VEGF), are now known biomarker between DM and GC. IGFBP family members have been shown to have a role in tumor formation and progression, and they may be valuable prognostic indicators in a variety of malignant tumors, including ovarian cancer [76], pancreatic cancer [77], and GC [78]. Currently, there is a scarcity of thorough research on IGFBP as a biomarker for GC.

Bioinformatics investigation reveals that IGFBP expression differs among GC cell lines and tissues [79]. IGFBP-1 is a blood biomarker with good diagnostic sensitivity for upper gastrointestinal cancer. Overexpression of IGFBP-1 inhibits MMP-9-induced GC cell migration and protects against *H. pylori*-induced GC [80]. Although clinical studies have shown that IGFBP-3 can be used as a biomarker for the diagnosis and prognosis of esophageal gastric junction adenocarcinoma [81] and that the simultaneous decrease of IGFBP-3 and increase of IGF-I may promote tumor growth [82], the mechanism underlying the relationship between these two potential biomarkers and GC has not been established. IGFBP-5 overexpression promotes the activity of the tumor suppressor factor PKNOX2, which can limit the development of GC [83]. IGFBP-7 mRNA expression is associated with a poor outcome in GC [84].

GC patients have a systemic biochemical imbalance of several growth factors, including notably raised levels of IGF-I and VEGF in advanced GC patients [85]. According to Saisana et al.'s findings, GC cells' survival depends on insulin receptors, insulin, and IGF signaling pathways that play a prominent role in gastric adenocarcinoma [73]. Higher IGF-IR expression is linked to a shorter overall survival. Serum IGF-I levels are considerably higher in patients with *H. pylori*-induced GC [86]. Upregulation of IGF-IR may activate the PI3K/AKT/mTOR signaling pathway, promoting GC cell migration and invasion [87].

VEGF is a critical proangiogenic factor that has emerged as the primary target of immunotherapy for GC [88]. Furthermore, animal models have demonstrated that IGFBP-4 can increase VEGF-induced angiogenesis [89] and that the m6A binding protein METTL3 can target VEGFA via IGFBP-2, encouraging the creation of colorectal cancer vasculogenic mimicry via the PI3K/AKT/mTOR and ERK1/2 signaling pathways [90]. Because IGF-IR regulates the production of VEGF ligands in GC cells and contributes to angiogenesis and lymphangiogenesis, inhibiting the IGF-I receptor can increase the antitumor impact of bevacizumab [91].

The interaction between numerous extracellular vesicles and immune-related cytokines released by GC cells [85], which are thought to be connected with the initiation and poor prognosis of GC [92], may be related to the imbalance of these growth factors. However, the peripheral concentration of growth factors does not have significant diagnostic potential. It cannot be utilized as an independent biomarker in patients to differentiate between different forms of GC. As a result, more experimental and clinical investigations, including other indicators, are required for validation.

### 3.4. Effect of *Helicobacter Pylori* Infection on DM and GC

*H. pylori*, a gram-negative, active, microaerobic, and spiral-shaped bacterium, is a major known risk factor for GC, and *H. pylori* infection is closely associated with more than 60% of GC cases [93]. Currently, the only natural host of *H. pylori* is the human stomach. The World Health Organization (WHO) has classified *H. pylori* as a Class I carcinogen [94]. *H. pylori* can cause oxidative stress and DNA damage through specific toxic cytokines such as cytotoxin-associated gene A (CagA), vacuolar cytotoxin A (VacA), and outer membrane protein and eventually lead to tumor formation [95]. Mucosal integrity can be compromised by phosphorylated CagA, which controls cytoskeleton and intercellular connections and their shape and function [96]. By turning on the carcinogenic YAP pathway, CagA also promotes GC's epithelial-mesenchymal transition [97]. Both CagA and VacA may induce autophagy [98, 99], and VacA is another virulence factor that can alter host cell metabolism by inhibiting mTORC1 [100].

Recent studies have demonstrated that *H. pylori* infection is closely related to DM and insulin resistance [101]. The creation of biofilms, decreased bacterial diversity, drastically reduced facultative anaerobic function, and increased abundance of *H. pylori* and *Haemophilus* are only a few of the important alterations in the stomach microbiota that can result from *H. pylori* infection [102]. Additionally, it was discovered that *H. pylori* corejected strongly with *Fusobacterium*, *Neisseria*, *Prevotella*, *Wechterella*, and *Roche* in patients with GC [103], and the gastrointestinal microbiota of these microorganisms would play a role in the pathogenesis of DM by controlling fatty acid synthesis and energy metabolism [104]. C.H. Tseng and F.H. Tseng found that patients with DM were shown to have a greater infection rate, a poorer eradication rate, and a higher reinfection rate [48]. *H. pylori* infection can lead to DNA damage by increasing the production of reactive oxygen species in epithelial cells of the gastrointestinal system, resulting in gastric mucosal atrophy, intestinal metaplasia, and, ultimately, the development of GC [105]. Ikeda et al. reported a significantly increased risk of GC in DM patients with *H. pylori* infection with baseline HBA1c levels higher than 6.0% [106]. Results of a large cohort study by Youn et al. showed that GC was associated with first-degree relatives with GC (OR = 3.23) in the absence of *H. pylori* and with hyperglycemia (OR = 1.98) in the presence of *H. pylori* [107]. However, according to Jun et al., there is no link between blood glucose and the risk of GC in either *H. pylori*-positive or *H. pylori*-negative DM patients [108]. In prediabetes patients, no correlation has been found between *H. pylori* infection and the risk of GC [109]. Interestingly, GC was shown to reduce the abundance of Helicobacter [110], *H. pylori* infection decreased with the progression of GC, and the diagnostic effectiveness of *H. pylori* decreased [111]. These contradictory findings imply that the impact of *H. pylori* infection on the risk of GC in DM individuals should be investigated further.

### 3.5. Effects of Gastric Microbiota (Other than *H. Pylori*) on DM and GC

Although successful *H. pylori* eradication does not completely prevent the development of GC and only about 1% of infected individuals develop GC [112], *H. pylori* infection plays a critical role in the early stages of carcinogenesis by increasing inflammation and gradually degrading gastric epithelial structure and function [113]. Additionally, compared to superficial gastritis, intestinal metaplasia and GC exhibit much lower levels of microbial diversity, which is now understood to be a characteristic of inflammatory illnesses and malignancies [114]. Some *Escherichia coli branched-chain proteins*, *Bacteroides fragilis*, *Clostridium nuclear*, and other pathogenic bacteria may contribute to the development of colorectal cancer [115]. Compared with the microbiota in chronic gastritis, the microbiota in GC patients not only increased the function of nitrite reductase, which promoted the reduction of nitrite to nitric oxide, but also increased the function of nitrite reductase, which promoted the reduction of nitrite to nitrite [112, 116]. Therefore, in addition to *H. pylori*, other gastric microorganisms may also contribute to the persistent inflammation of gastric mucosa and the development of GC, including *Citrobacter*, *Clostridium*, *Lactobacillus*, *Achromobacter*, and *Rhodococcus*, which reside in the intestinal mucosa as commensals [117].

All of these findings suggest that the mechanism by which bacteria promote tumor growth may be connected to producing an inflammatory response and altering host immunological function [118]. The immune system is an essential regulator that promotes or inhibits tumor biological function [119], and the intestinal microbiota can drive immune system development and function [120], as well as alter intestinal function and immune system [121]. More and more research suggests that the gastrointestinal symbiotic microflora can modulate host immunity and maintain host immunological homeostasis. For example, in the GC microenvironment, the amount of BDCA^2+^ plasmacytoid dendritic cells is positively connected with the number of stenotrophomonas, whereas the number of Foxp^3+^ regulatory T cells is strongly correlated with the number of selenodont [122]. An imbalance in the microorganisms of the intestine promotes the establishment of an immunosuppressive microenvironment [123]. AMP, IgA, ROC, and phagocytosis are ways the immune system modulates the microbiota. In turn, the microbiome creates compounds that control immune system activity [124].

Although the influence of *H. pylori* infection on the incidence of GC in DM individuals is still debated, alterations in gastrointestinal microbiota other than *H. pylori* have been linked to DM, and these gastrointestinal microbiota are thought to be key players in the interaction between *H. pylori* infection and metabolic diseases such as DM [125]. The study discovered abnormalities in the gastrointestinal microbiota of DM. Among the commonly reported findings, the genera of *A. muciniphila* [126, 127], *Bifidobacterium* [128], *Bacteroides* [129], *Faecalibacterium* [130], *F. prausnitzii* [131], *C. leptum* [132], *Oscillospiracea*e [126], and *Akkermansia* [133] were negatively associated with T2DM, while the genera of *Ruminococcus* [131, 134], *Dorea* [135], and *Blautia* [126] were positively associated with T2DM. These microbiome alterations influence inflammation, glucose and lipid metabolism, insulin sensitivity, and overall energy balance [135, 136]. For example, lipopolysaccharides, as a product of gastrointestinal microbiota, can promote metabolic endotoxemia and low-grade inflammation [137], and *Roseburia intestinalis*, *Bacteroides fragilis*, *Akkermansia muciniphila*, *Lactobacillus plantarum*, and *L. casei* can stimulate the production of anti-inflammatory cytokines and chemokines [138]. *R. intestinalis* can increase T regulatory cell development, stimulate TGF-*β*, and suppress intestinal inflammation [139]. *Bacteroides* also increased gene expression in T regulatory cells [140]. *L. plantarum*, *L. paracasei*, and *L. case* can decrease IL-1*β*, monocyte chemoattractant protein-1, intercellular adhesion molecule-1, IL-8, CD36, and C-reactive protein [141]. *Lactobacillus* [142] and *Akkermansia* [143] have been found to suppress TNF-*α*. *L. paracasei* and microbial anti-inflammatory molecule from *F. prausnitzii* inhibit the activity of NF-*κ*B [144]. As a metabolic product of gastrointestinal microbiota, short-chain fatty acids (SCFA) can not only directly prevent low-grade inflammation and enhance the secretion of glucagon-like peptide 1 (GLP-1) but also increase insulin sensitivity and affect cell function and insulin secretion [145].

Changes in the composition, variety, and activity of the microbiota can cause a disruption in glucose metabolism, which is a key factor in the development of T2DM [146]. *Bifidobacterium lactis* can both boost glycogen production and decrease the expression of gluconeogenesis-related genes in the liver, such as glucose-6-phosphatase and phosphoenolpyruvate carboxykinase [147]. It can also improve endotoxin-related inflammation and impaired intestinal barrier function, perhaps with antidiabetic benefits [148]. *Lactobacillus butyrate* reduces insulin resistance in the liver by raising mRNA levels of PI3K, insulin receptor substrate-2, AMPK, Akt2, and glycogen production [149]. *Lactobacillus tyrosine* also lowers blood sugar levels via the cholic acid-chlorine exchange pathway [150]. Furthermore, certain gastrointestinal bacteria can promote fatty acid oxidation and energy expenditure while decreasing fatty acid synthesis, improving T2DM, such as *Akkermansia muciniphila*, *Bacteroides acidifaciens*, *Lactobacillus gasseri*, and SCFA [136]. Moreover, the products of these microorganisms, such as butyrate, can promote fatty acid oxidation and thermogenesis by inhibiting the histone deacetylation process in the muscle, thereby increasing energy expenditure by promoting mitochondrial function in the muscle [151].

In conclusion, a diverse gastrointestinal microbiota is critical for general metabolic health. The intestinal microbiota may be a crucial regulator of host glucose metabolism and immune response. When the microbiota is out of balance, it can contribute to pathological processes such as GC and DM. However, the gastrointestinal microbiota is a complex ecosystem, and further study is needed to determine which microorganisms are responsible for the pathophysiology and molecular processes of GC and DM.

### 3.6. Effect of Metformin on DM and GC

Metformin, used as a first-line medicine in the treatment of DM, has a direct anticancer impact on a wide variety of tumor cells, including tumor stem cells, in both insulin-dependent and insulin-independent models [152]. It can not only promote the expression of metabolic checkpoints related to T cells and immunosuppressive cells in the tumor environment in cancer cells [153, 154] but also has systemic impacts on metabolism by interfering with gastrointestinal microbiota [155]. *In vitro* and *in vivo* model studies have shown that in digestive system cancers, metformin provides chemoprophylactic effects and direct therapeutic action [156], and it has the potential to be a chemical and radiosensitizer, increasing the sensitivity of cancer cell lines to 5-fluorouracil (5-FU) and paclitaxel [157]. Most clinical studies have demonstrated that metformin can reduce the risk of gastrointestinal cancer and improve survival rates [158]. However, there is no solid evidence showing that metformin usage increases the risk of GC [159, 160].

Metformin has been proven to protect against GC in various observational studies in recent years [161, 162] (Table 2). Cheung et al. showed that metformin can reduce GC risk (HR = 0.49, 95% CI: 0.24-0.98), which decreases further with increasing dose and duration [162]. Tseng also demonstrated that metformin reduces GC risk, especially when the cumulative duration exceeds 2 years [163]. Another meta-analysis also showed a 21% reduction in GC risk with the use of metformin (HR = 0.790; 95% CI: 0.624-1.001), especially in Asian populations [164]. Metformin was shown to minimize GC recurrence in gastrectomy patients in two retrospective investigations [165, 166]. Despite this, some observational studies in the USA [167], the Netherlands [168], and Sweden [169] did not show a lower risk of GC associated with metformin use. Whether metformin can improve the prognosis of GC in patients with DM remains controversial. In the study of Dulskas et al., although metformin was associated with a reduced risk of GC, it did not affect the survival rate of patients with DM and GC [170, 171]. The studies conducted by Baglia et al. and Chen et al. did not similarly observe the survival benefits of metformin for GC [172, 173]. In contrast, metformin improved overall survival but not cancer-specific survival. Studies conducted by Lacroix et al. [174], Seo et al. [166], and Chung et al. [175] showed that metformin can improve the survival rate of patients with T2DM and reduce GC recurrence.

Most previous clinical studies have been retrospective and often limited by immortal time bias and selection bias, and the link between metformin use and GC risk has been exaggerated [176]. More clinical research is needed to validate the role of metformin in the treatment and chemoprophylaxis of GC. In particular, in *in vitro* and *in vivo* studies on metabolism and cell cycle arrest, possible therapeutic targets for metformin have been identified to enhance the anticancer effects of chemotherapy by regulating inflammation. In tumor xenograft models, metformin alone decreased tumor volume, and cisplatin, rapamycin, or both boosted the impact of each treatment alone and blocked GC cell peritoneal spread [177]. *In vitro* studies have shown that combining metformin with cisplatin, adriamycin, and paclitaxel may improve the unique effects of each treatment, and the combination with the three chemotherapy drugs can effectively induce the apoptosis of AGS cells [178]. However, the biological mechanism of this association remains unclear; several possible mechanisms could explain the protective effect of metformin. First, metformin directly activates AMPK and inhibits cell proliferation by inhibiting cancer-related central signaling pathways, such as the PI3K/Akt/mTOR pathway [179]. Second, metformin-induced decreases in IGF concentrations in circulating insulin may lower activation of IGF/IGF1-R signaling, resulting in reduced growth promotion and mitogenesis [180]. As a result, the anticancer effects of metformin may be attributed to its capacity to modify the metabolic milieu or to directly act on tumor cells. Third, the significant intracellular metabolic changes induced by metformin are the reduced accumulation of glycolytic intermediates and the synergistic reduction in tricarboxylic acid cycle intermediates, contributing to a reduction in gluconeogenesis [181]. The activation of AMPK promotes glucose uptake in fat and muscle, inhibiting tumor cell proliferation and migration [182]. Fourth, the protective effect of metformin may be related to the inhibition of HIF1*α*/PKM2 signal transduction [183]. Metformin induces the downregulation of hypoxia-inducible factor 1*α* and TNF-*α*, which can inhibit angiogenesis and improve immune surveillance by reducing tumor hypoxia [184]. Finally, studies in recent years have suggested that metformin may have a potential protective effect against *H. pylori* infection. After eradication of *H. pylori*, metformin reduced the risk of GC by 51% in DM patients [138]. On the one hand, the persistent inflammatory response brought on by *H. pylori* colonization is the strongest single risk factor for GC [185]. Metformin plays an anti-inflammatory role by inhibiting cell signaling pathways and reducing the production of proinflammatory factors [186], which can reduce the inflammatory response brought on by *H. pylori*. On the other hand, metformin can regulate the function of the immune system, including enhancing the activity of natural immune cells and regulating the immune response [187]. In addition to improving the body's resistance to *H. pylori* infection, metformin can also enhance the effectiveness of cancer treatments, though the molecular mechanisms underlying these effects are not fully understood [188]. Recent research has also demonstrated that metformin can not only regulate gastrointestinal microbiota in composition and function to enhance its glucose-regulating effect [189] but also promote gastric acid secretion by activating AMPK to differentiate gastric epithelial progenitor cells into acid-secreting parietal cells [190], thereby alleviating the reduction in gastric acid secretion brought on by *H. pylori* infection [116]. This enhances metformin's glucose-regulating effect. Metformin may have the potential to be an anti-GC medication by encouraging the differentiation of gastric epithelial progenitor cells into acid-secreting parietal cells [191], which is thought to be important in *H. pylori* infection and the occurrence and development of GC [192].

The changes in intracellular pathways caused by tumorigenesis and the underlying mechanism of the antitumor activities of metformin have been confirmed, revealing new therapeutic targets. However, these are not the only treatments available to reduce cancer risk. Insulin resistance, DM, the chronic diseases associated with inflammation in the microenvironment, and specific tumor-driven oncogenic pathways may interfere with the direct and indirect antitumor effects of metformin. Although epidemiological in nature, *in vivo* and *vitro* studies and clinical data support the benefit of metformin in some patients with digestive tumors, but survival outcomes are influenced by a variety of factors, such as cancer type, differentiation, staging, and treatment. Therefore, to fully understand metformin use in gastrointestinal tumors, rigorous clinical trials are needed to identify patients who may benefit from metformin.

## 4. Conclusions

DM is linked to an increased risk of cancer and cancer-related mortality, and the association between DM and GC is inconclusive. The positive association reported in most published meta-analyses suggests that DM may be an independent risk factor for GC, regardless of statistical significance. Potential mechanisms may include hyperinsulinemia, insulin resistance, elevated IGF-I levels, oxidative stress, chronic inflammation, and anti-insulin medication use. Activation of these signaling pathways is responsible for the development of GC in DM patients. Understanding the relationship between DM and GC may provide novel therapeutic strategies to counter the poor prognosis caused by this correlation.

## Figures and Tables

**Table 1:** Remission of DM after gastrectomy.

Author	Surgery	Sample size	CR	PR	Follow-up (mo)
Lee et al. [34]	RYTG, BI, BII, RYGJ	229	19.70%	37.10%	NA
An et al. [35]	RYTG, BI, BII	64	3.10%	54.70%	12
Kim et al. [36]	RYTG, BI, BII	385	15.10%	30.40%	33.7
Choi et al. [38]	RYTG, BI	40	2.50%	40.00%	12
Wei et al. [40]	RYTG, BII	67	26.90%	32.80%	57.4

CR: complete remission; PR: partial remission; RYTG: Roux-en-Y total gastrectomy; BI: Billroth I reconstruction; BII: Billroth II reconstruction; RYGJ: subtotal gastrectomy with Roux-en-Y gastrojejunostomy reconstruction; NA: not available.

**Table 2:** Clinical studies of metformin for the treatment of GC.

Author	Study design	Inclusion criteria	HR
Tseng [163]	Retrospective cohort study	DM2 + antidiabetic drugs	HR: 0.45 (0.36-0.56)
Lee et al. [165]	Retrospective cohort study	GC + gastrectomy	HR: 0.58 (0.37-0.93)
de Jong et al. [168]	Retrospective cohort study	DM2 + oral antidiabetic drugs	HR: 0.97 (0.82-1.15)
Zhou et al. [183]	Meta-analysis, 7 cohort studies	GC + metformin	HR: 0.76 (0.64-0.91)
Lacroix et al. [174]	Retrospective cohort study	GC	HR: 0.86 (0.56-1.33)
Zheng et al. [169]	Prospective cohort study	DM2 + antidiabetic drugs	Noncardia: HR: 0.93 (0.78-1.12). Cardia: HR: 1.49 (1.09-2.02)
Baglia et al. [172]	Prospective cohort study	Breast, CRC, lung, and GC patients	OS-HR: 1.11 (0.81-1.53)
Seo et al. [166]	Retrospective cohort study	GC + curative gastrectomy	HR: 0.45 (0.30-0.66)
Dulskas et al. [170]	Retrospective cohort study	DM2 + GC	SIR: 0.75 (0.66-0.86)
Shuai et al. [164]	Meta-analysis, 11 cohort studies	GC + metformin	HR: 0.79 (0.62-1.00)

## Data Availability

The data used to support the findings of this study are included in the article.
